# Supplementary material for: Investigating the Effects of Non-Branded Foods Placed in Cartoons on Children’s Food Choices through Type of Food, Modality and Age
Source: Int J Environ Res Public Health. 2019 Dec 10;16(24):5032. doi: 10.3390/ijerph16245032 (PMC6950664; doi:10.3390/ijerph16245032)
Supplement: Supplementary file 1 [file ijerph-16-05032-s001.pdf]

International Journal of
Environmental Research and Public Health

MDPI

Article

Investigating the Effects of Non-Branded Foods Placed in Cartoons on Children's Food Choices through Type of Food, Modality and Age

Foods cards distribution

Bimodal low recommended foods

Bacon

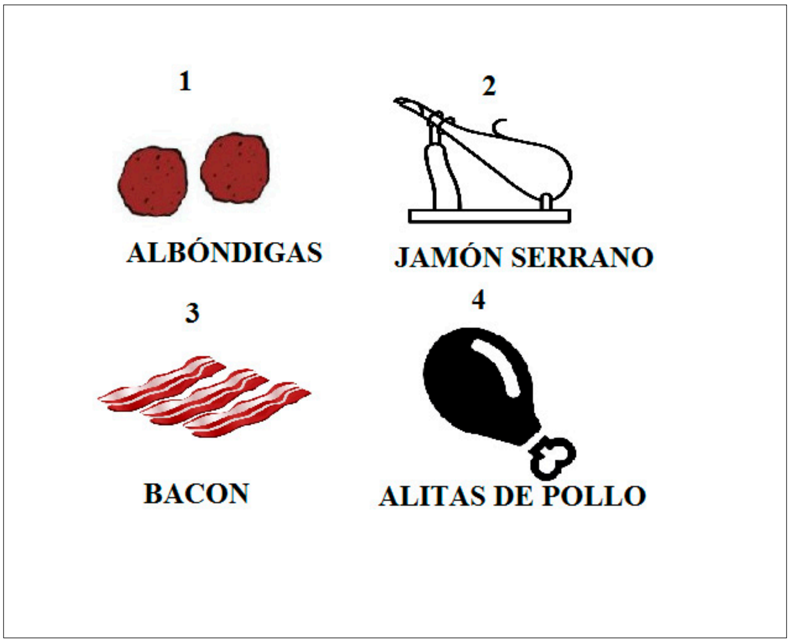

Burrito

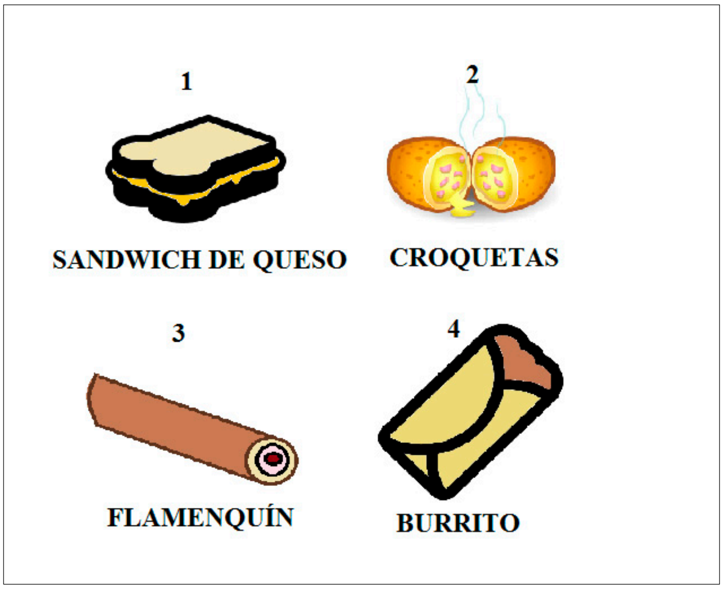

Gummy bears

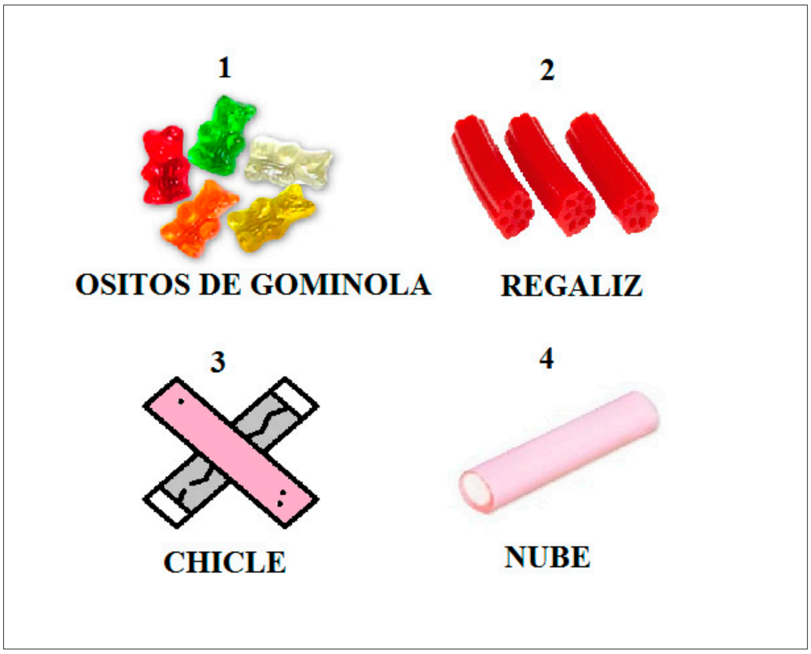

Mayonnaise

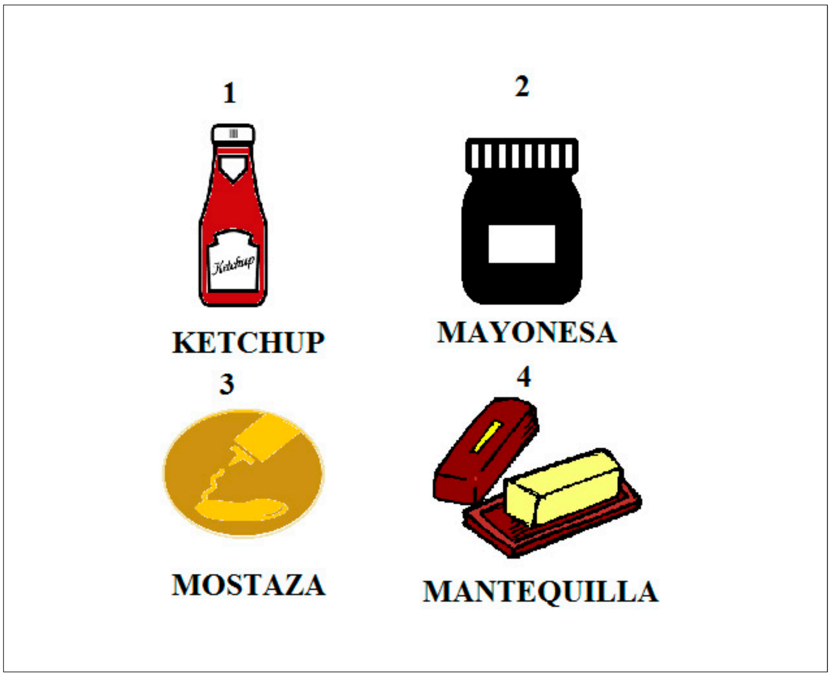

Unimodal low recommended foods

Cookies

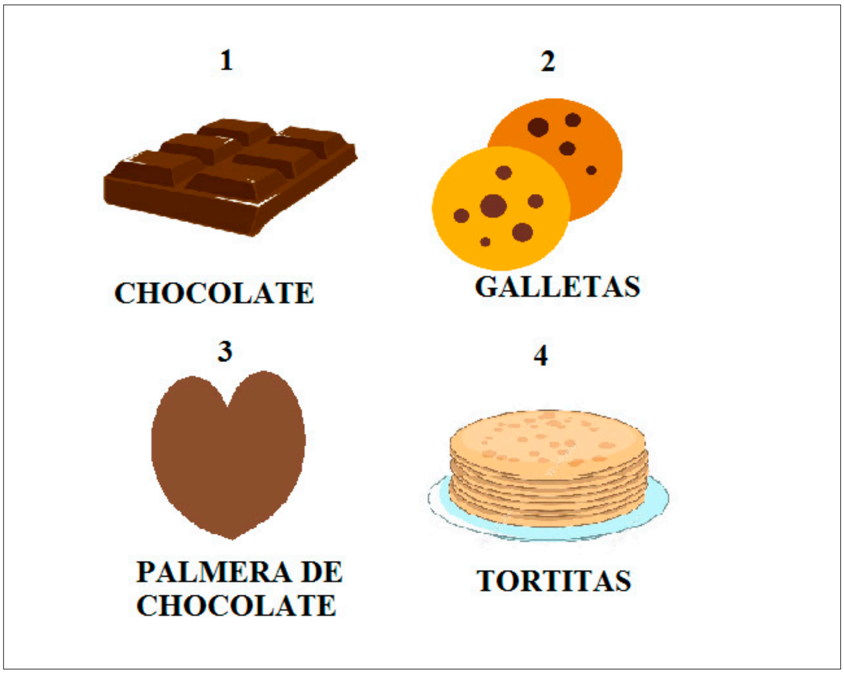

Hot dog

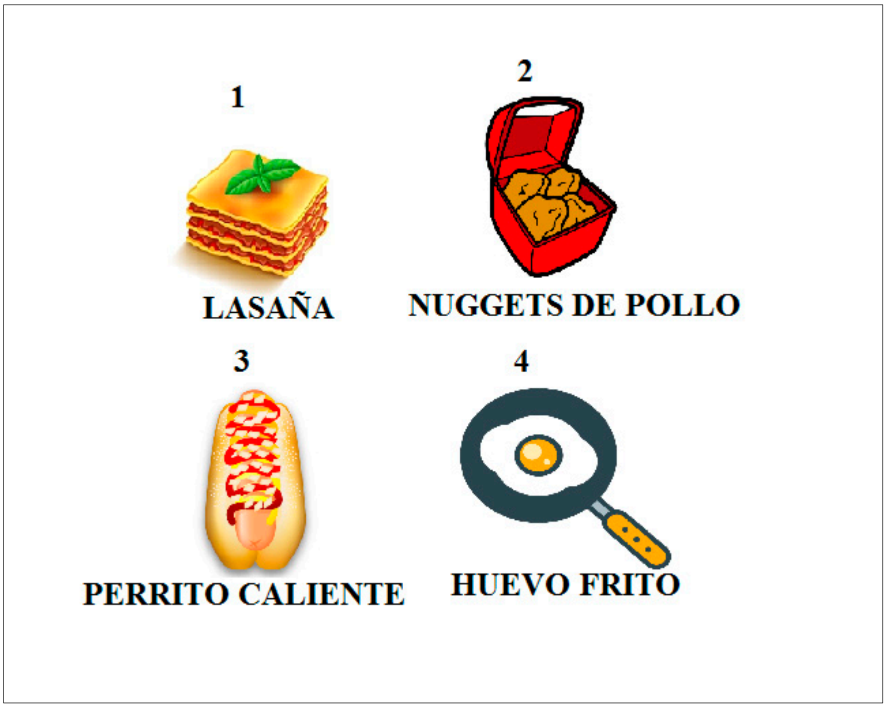

Nachos

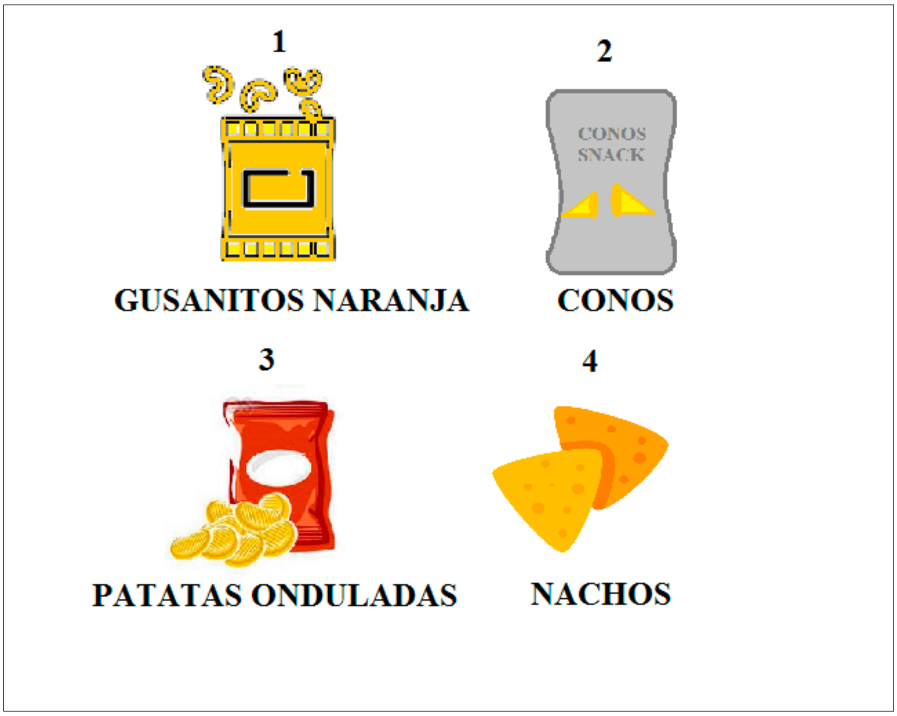

Pizza

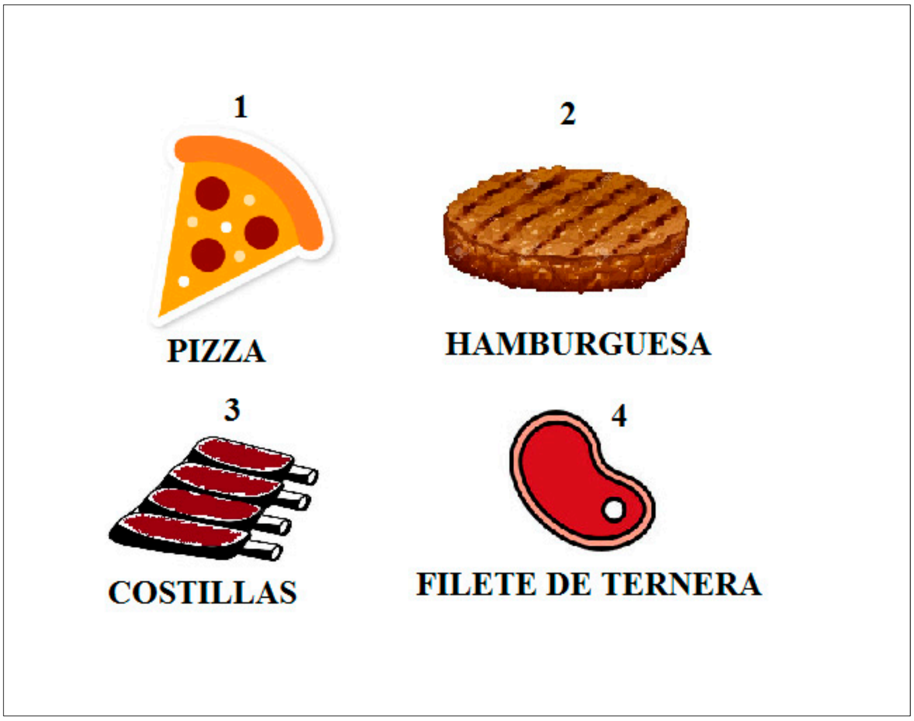

Bimodal highly recommended foods

Grapes

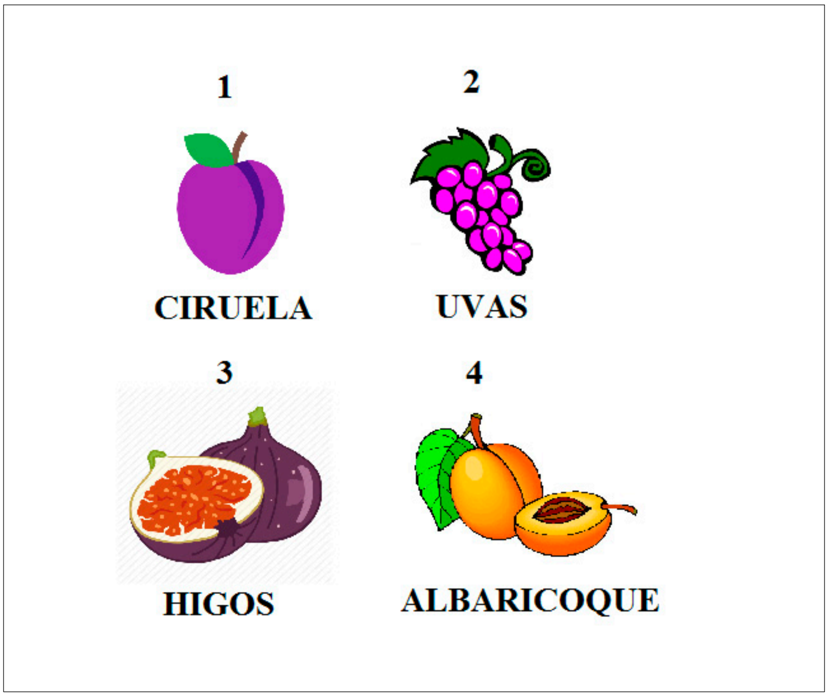

Lettuce

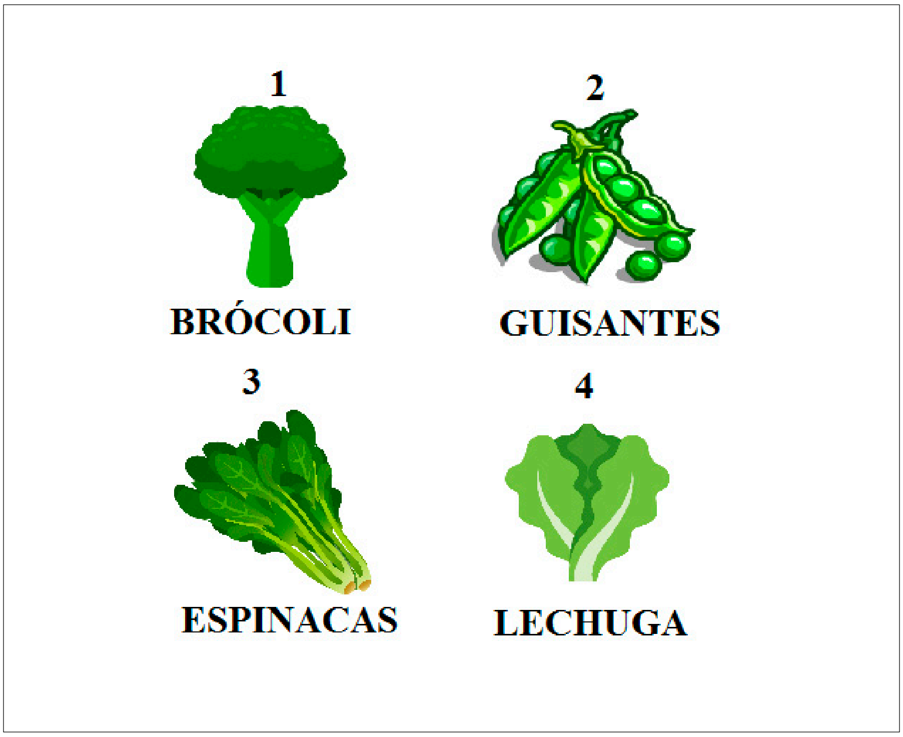

Pineapple

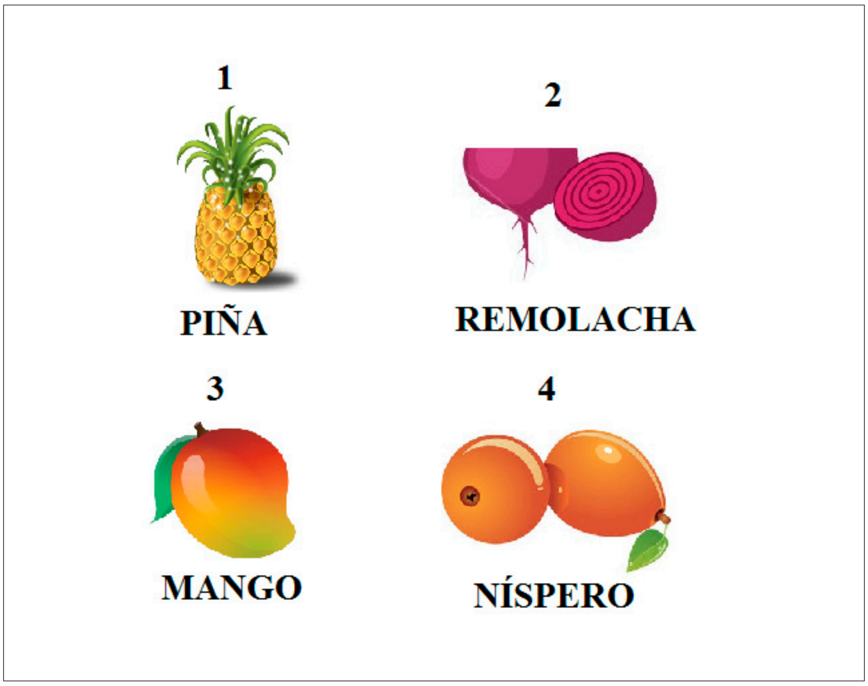

Toast

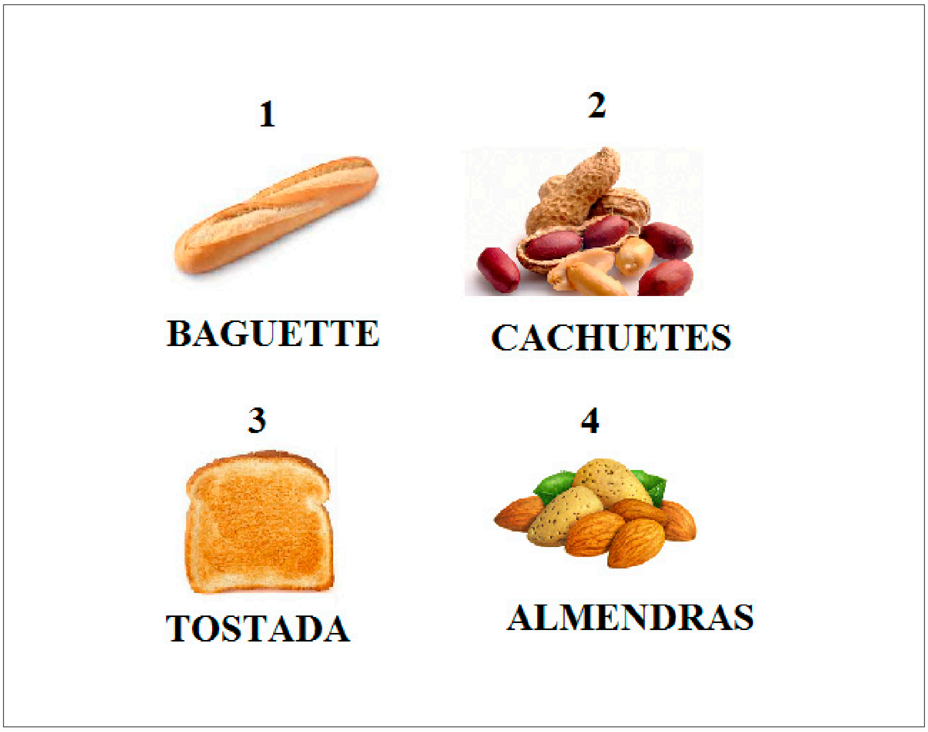

Unimodal highly recommended foods

Asparagus

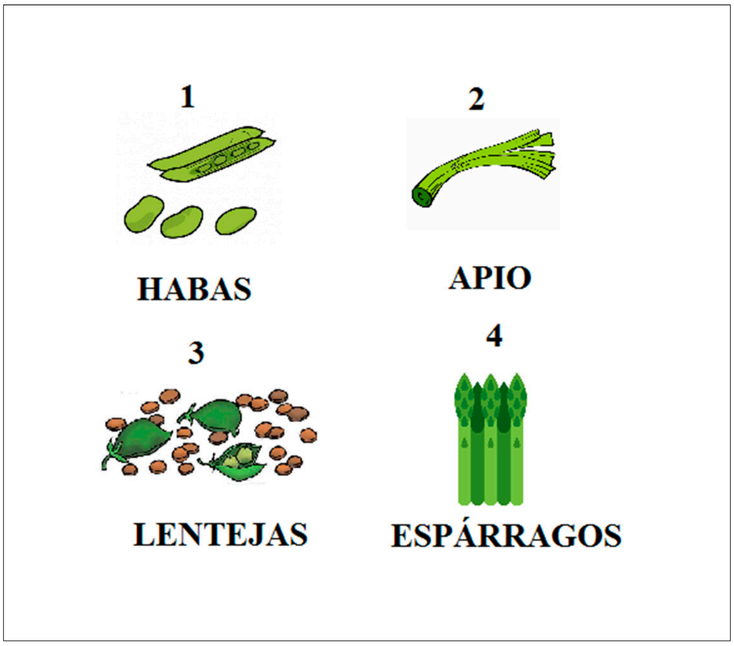

Coconut

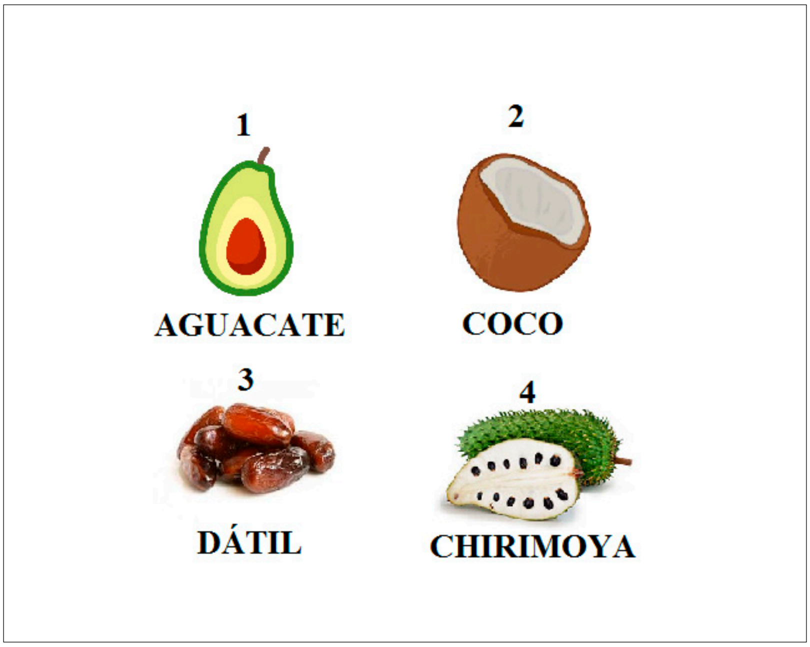

Corn

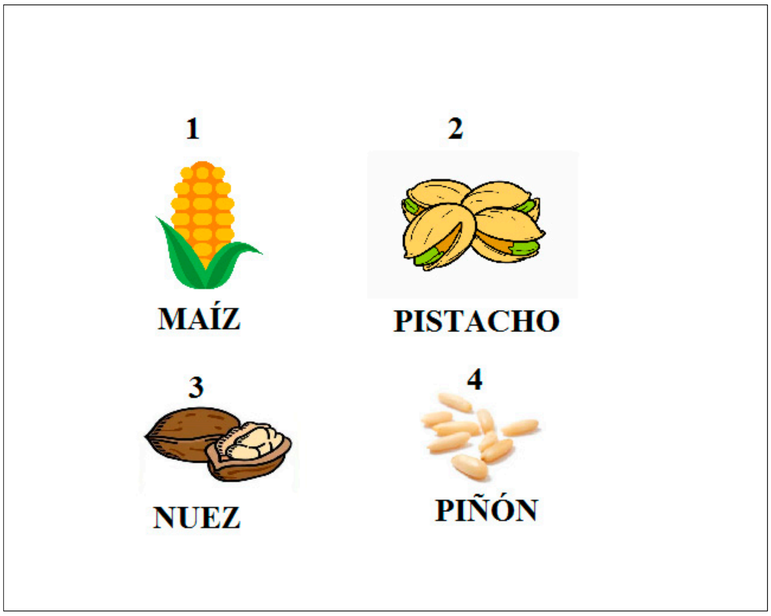

Water

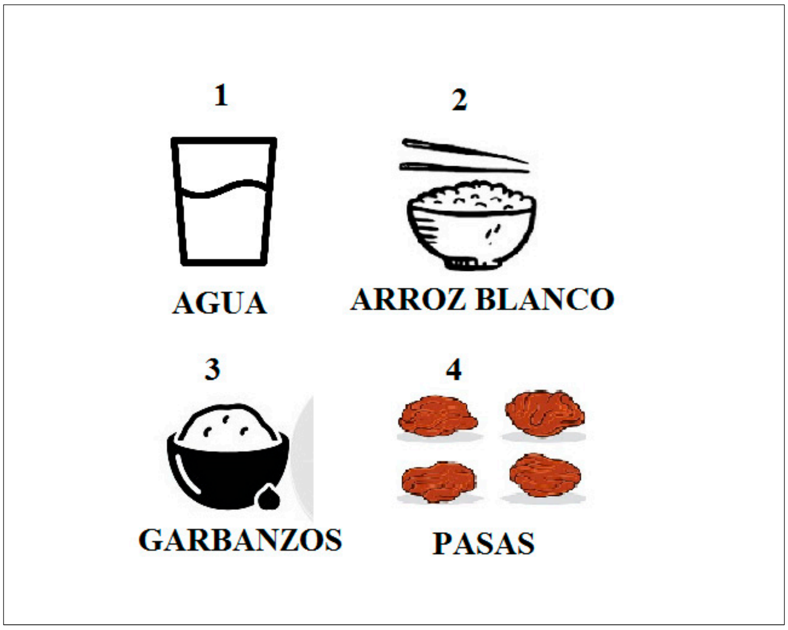

© 2019 by the authors. Submitted for possible open access publication under the terms and conditions of the Creative Commons Attribution (CC BY) license (http://creativecommons.org/licenses/by/4.0/).
